# Recruiting participants for focus groups in health research: a meta-research study

**DOI:** 10.1186/s12874-025-02464-x

**Published:** 2025-01-14

**Authors:** Jonas Lander, Simon Wallraf, Dawid Pieper, Ronny Klawunn, Hala Altawil, Marie-Luise Dierks, Cosima John

**Affiliations:** 1 Hannover Medical School (MHH), Institute for Epidemiology, Social Medicine and Health Systems Research, Carl-Neuberg-Street 1, 30625 Hannover, Germany; 2 Faculty of Health Sciences Brandenburg, Brandenburg Medical School Theodor Fontane, Institute for Health Services and Health System Research, Rüdersdorf, Germany; 3 Center for Health Services Research Brandenburg, Brandenburg Medical School Theodor Fontane, Rüdersdorf, Germany

**Keywords:** Focus groups, Study participants, Recruitment, Recruiting, Health research, Participant selection, Qualitative methods

## Abstract

**Background:**

Focus groups (FGs) are an established method in health research to capture a full range of different perspectives on a particular research question. The extent to which they are effective depends, not least, on the composition of the participants. This study aimed to investigate how published FG studies plan and conduct the recruitment of study participants. We looked at what kind of information is reported about recruitment practices and what this reveals about the comprehensiveness of the actual recruitment plans and practices.

**Methods:**

We conducted a systematic search of FG studies in PubMed and Web of Science published between 2018 and 2024, and included *n* = 80 eligible publications in the analysis. We used a text extraction sheet to collect all relevant recruitment information from each study. We then coded the extracted text passages and summarised the findings descriptively.

**Results:**

Nearly half (*n* = 38/80) of the studies were from the USA and Canada, many addressing issues related to diabetes, cancer, mental health and chronic diseases. For recruitment planning, 20% reported a specific sampling target, while 6% used existing studies or literature for organisational and content planning. A further 10% reported previous recruitment experience of the researchers. The studies varied in terms of number of participants (range = 7–202) and group size (range = 7–20). Recruitment occurred often in healthcare settings, rarely through digital channels and everyday places. FG participants were most commonly recruited by the research team (21%) or by health professionals (16%), with less collaboration with public organisations (10%) and little indication of the number of people involved (13%). A financial incentive for participants was used in 43% of cases, and 19% reported participatory approaches to plan and carry out recruitment. 65 studies (81%) reported a total of 58 limitations related to recruitment.

**Conclusions:**

The reporting of recruitment often seems to be incomplete, and its performance lacking. Hence, guidelines and recruitment recommendations designed to assist researchers are not yet adequately serving their purpose. Researchers may benefit from more practical support, such as early training on key principles and options for effective recruitment strategies provided by institutions in their immediate professional environment, e.g. universities, faculties or scientific associations.

**Supplementary Information:**

The online version contains supplementary material available at 10.1186/s12874-025-02464-x.

## Background

The recruitment of study participants is an essential task for health research, as the process and outcomes of study participation are impacted by who participates and how [1, 2]. However, the search for participants raises various questions and challenges: several reviews summarise the reasons for and against study participation and key success factors, e.g. that participation is more likely if the invitation is made personally, or that recruitment can be improved when recruitment sites other than health care settings are used [3, 4]. The use of incentives [5] and the effectiveness of social media recruitment [6, 7] have also been systematically analysed. Regarding incentives, it has been shown that, prior to conducting a study, there needs to be clarity in terms of which incentives to use, how and for what reason and for which target group in order to be effective [5]. For instance, potential participants with a higher socio-economic status may not be sufficiently motivated by a financial incentive.

Further, interview studies have been conducted with those who are directly involved in recruitment, in particular research project leaders [8] and those who often approach people for studies due to their professional position, such as doctors and nurses [9]. It has been shown that health professionals find it difficult to make people aware of research participation on top of their regular work [10]. In addition, qualitative and quantitative studies have evaluated the actual success of the recruitment methods reported (e.g. [11]).

Due to the variety of requirements and challenges, there are various ‘recruitment frameworks’, most of which were developed to provide guidance for researchers [3, 12]. In addition, reporting guidelines — here, the Consolidated Criteria for Reporting Qualitative Research (COREQ) [13] and the Standards for Reporting Qualitative Research (SRQR) [14] — provide guidance for researchers not just about which elements of a study to mention in a publication, including recruitment, but also on recruitment planning, e.g. how to approach potential study participants.

One methodological approach to empirical data collection in which recruitment is important is focus groups (FGs). FGs are a qualitative research method to capture the range of opinions, experiences and perspectives on specific health-related research questions [15]. The approach originated from social and market research, and, since its evolution in the 1940s, is being applied in various fields, including psychology, communication science, and health research [16]. In comparison to qualitative individual interviews, for example, similarities and differences in experiences or arguments can be identified based on the exchange and discussion between the participants [17]. At the same time, participants are encouraged to perceive and verbalise aspects that were previously only subconscious and/or tacit. This is crucial in health research, as true preferences, needs and beliefs may only emerge when arguments and ideas are exchanged [18]. The focus is on analysing participants’ views and experiences with health and disease-related issues in the context of a discussion topic [19].

In this sense, FGs can provide rich data to uncover similarities and differences among group participants [20]. Some methodological aspects of study execution itself have been investigated for focus groups, such as the role of the moderator or the transition from analogue to digital interview formats and corresponding instructions [21] [22], as well as guidelines for methodological reporting (e.g. [13]). Research in German-speaking countries has already shown that some relevant aspects, e.g. how participants really interact during the discussion, are not sufficiently addressed in publications, assuming that there is as yet no comprehensive, ‘best practice’ of focus group studies [23].

However, the extent to which FGs are effective depends not only on basic organisational and content issues [24], but also on the selection and composition of participants. In turn, if a sample is ‘well composed’, this can contribute to the quality of the collected data, because e.g. a diverse sample may provide more nuanced perspectives on a given topic. However, there are few insights about how recruitment for FGs actually takes place. While summarizing e.g. recruitment challenges or providing recruitment guidance is important, a clearer understanding of current practice would contribute to a better evidence base for any suggested measures.

The objective of this study was to investigate how published FG studies in health research planned and conducted recruitment of study participants. By examining the reported information, we aimed to deepen the understanding of participant recruitment, including the extent of recruitment efforts, and to gain further insight into how these efforts are reported. This dual focus provides a comprehensive view of recruitment and reporting practices in published studies. Our findings may help researchers refine their recruitment strategies and improve reporting for future studies.

## Methods

### Conceptual background

In our study, we concentrate on recruitment processes in the context of focus group studies, which is a methodological aspect of this research. To do so, firstly, it was necessary to understand which aspects of recruitment actually matter during the planning and conduct of respective processes. Since there is no uniform, agreed framework or guideline for how recruitment should be done, we summarized relevant aspects from two types of sources. Firstly, we searched the above-mentioned reporting guidelines for qualitative research (COREQ, SQRQ) for guidance on what should be reported regarding “participant selection”, i.e. who was approached, with what aim, via which channels, when/for how long the recruitment was done and by whom [13, 14]. We used relevant information from these checklists as the basis for our study — particularly, to specify which data we extracted from published focus group studies (see below). Secondly, we searched for studies that provide recommendations for researchers regarding which aspects may increase effectiveness and quality of the recruitment process [3, 4, 11, 25–27]. We summarized the various aspects mentioned in these studies — e.g. the importance of pre-estimating the time required and experience of the research team with recruitment should be estimated prior to recruitment, the precise approach to make potential participants aware of the study, and the active engagement of participants beyond answering questions, completing surveys etc. — and then, re-framed/shortened them into actual, concrete assessment items.

Based on the overview of the aspects found in the reporting checklists as well as in the recruitment literature, we agreed a list of categories/domains for further investigation (characteristics of study participants, characteristics of recruiters, recruitment planning and conduct, strategies to account for recruitment quality; see also data extraction). As described above, these aspects can be considered aspects of ‘reporting’, but they also matter in terms of recruitment ‘best practice’, for instance when considering which recruitment channel(s) might be more or less effective.

We explored these aspects via a meta-research approach (see e.g. [28]), since this study aims to do research on how research, i.e. the recruitment of FG-study participants is done, in order to suggest what kind of changes or improvements may be needed. Further, we used a systematic literature search to identify relevant focus group studies, which we report based on the PRISMA 2020 Statement [29], where applicable.

### Eligibility criteria

We first defined our inclusion criteria for the systematic selection of relevant studies (Table 1): FG studies with adult participants who are not healthcare professionals. We focused on the recruiting of the lay people/patients 1) to increase comparability within our sample by focusing on a more homogeneous group and 2) as currently available literature often associates recruitment challenges and planning complexities with this group, as opposed to healthcare professionals (e.g. doctors, nurses) who are generally more familiar with research participation (e.g. [30, 31]). We also excluded online FGs, as recruitment for digital data collection methods may require a separate investigation. Studies from health-related research were included — we identified relevant studies using the search terms outlined in Additional file 1. The number of focus groups conducted in the studies had no influence on their selection. To ensure that the included studies were reasonably up to date, we set the publication period to 2018–2024. We also restricted the search to studies published in English or German, the languages spoken by the research team.

**Table 1:** Inclusion criteria

1	The full publication of the article is available.
2	The language of publication is German or English.
3	The article was published between January 2018 – October 2024.
4	The article is not a multiple publication.
5	*Study design* Inclusion: qualitative studies with one or more focus groups. Exclusion: mixed-methods studies (e.g. studies with focus groups and a quantitative survey), online FG, meta-analyses, RTCs, CTs, reviews, case studies, case reports, conference papers, editorials, letters and grey literature.
6	*Participants* Inclusion: Studies with participants with a minimum age of 18 years. Exclusion: Focus group studies with underage participants and/or healthcare professionals. Publications that list mixed groups of participants, e.g. patients and health professionals.

### Information sources and search strategy

The systematic literature search was conducted in the bibliographic databases PubMed and Web of Science. The search strategy was first developed for PubMed and then adapted for Web of Science. It consists of two thematic blocks:

1. the first block relates to the field of health science research and includes terms such as ‘public health systems research’ and ‘health services research’.
2. the second block focusses on the design of focus group studies with search terms such as ‘focus groups’ or ‘group discussion’.

**Fig. 1:**
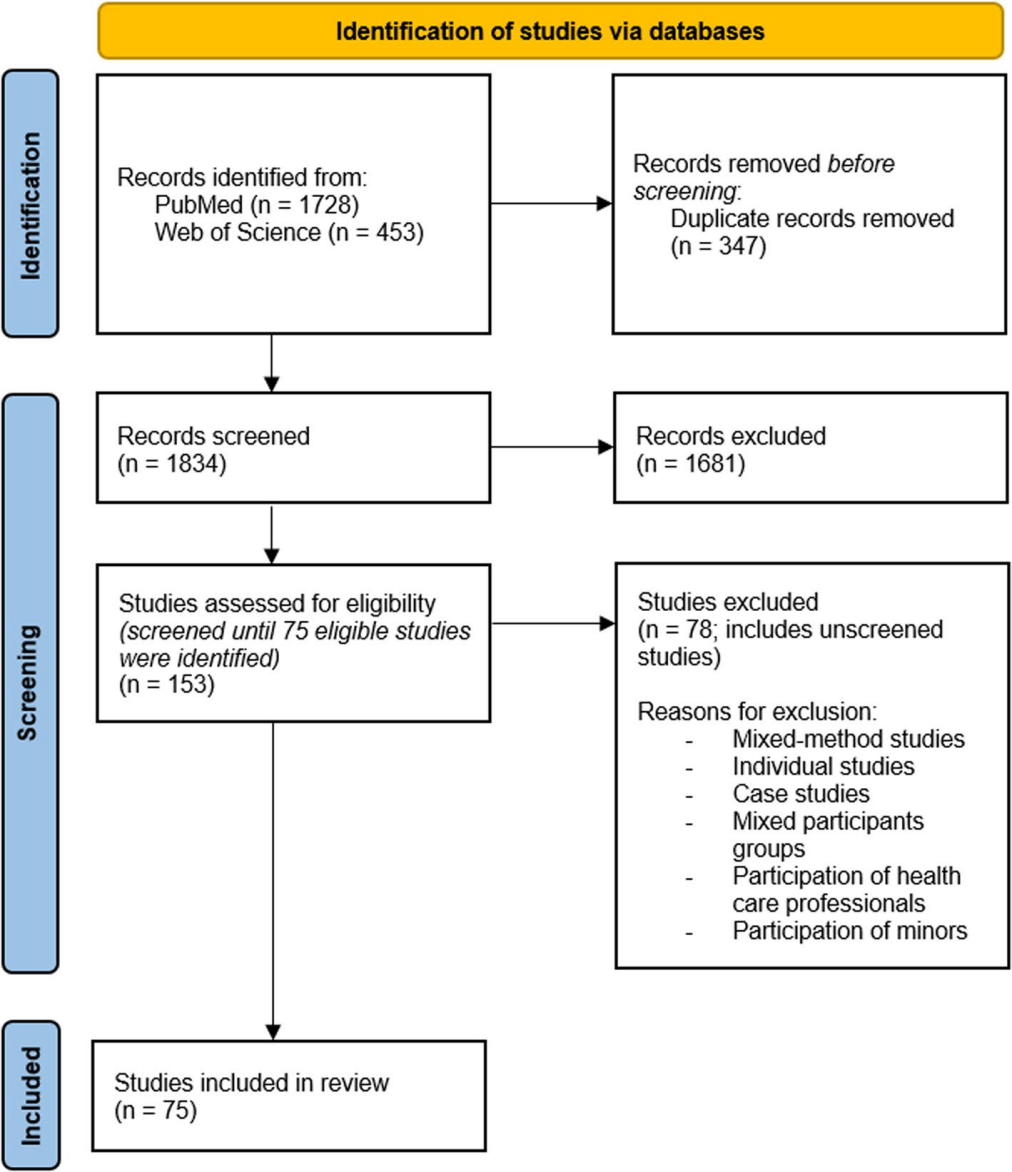
PRISMA flowchart

**Fig. 2:**
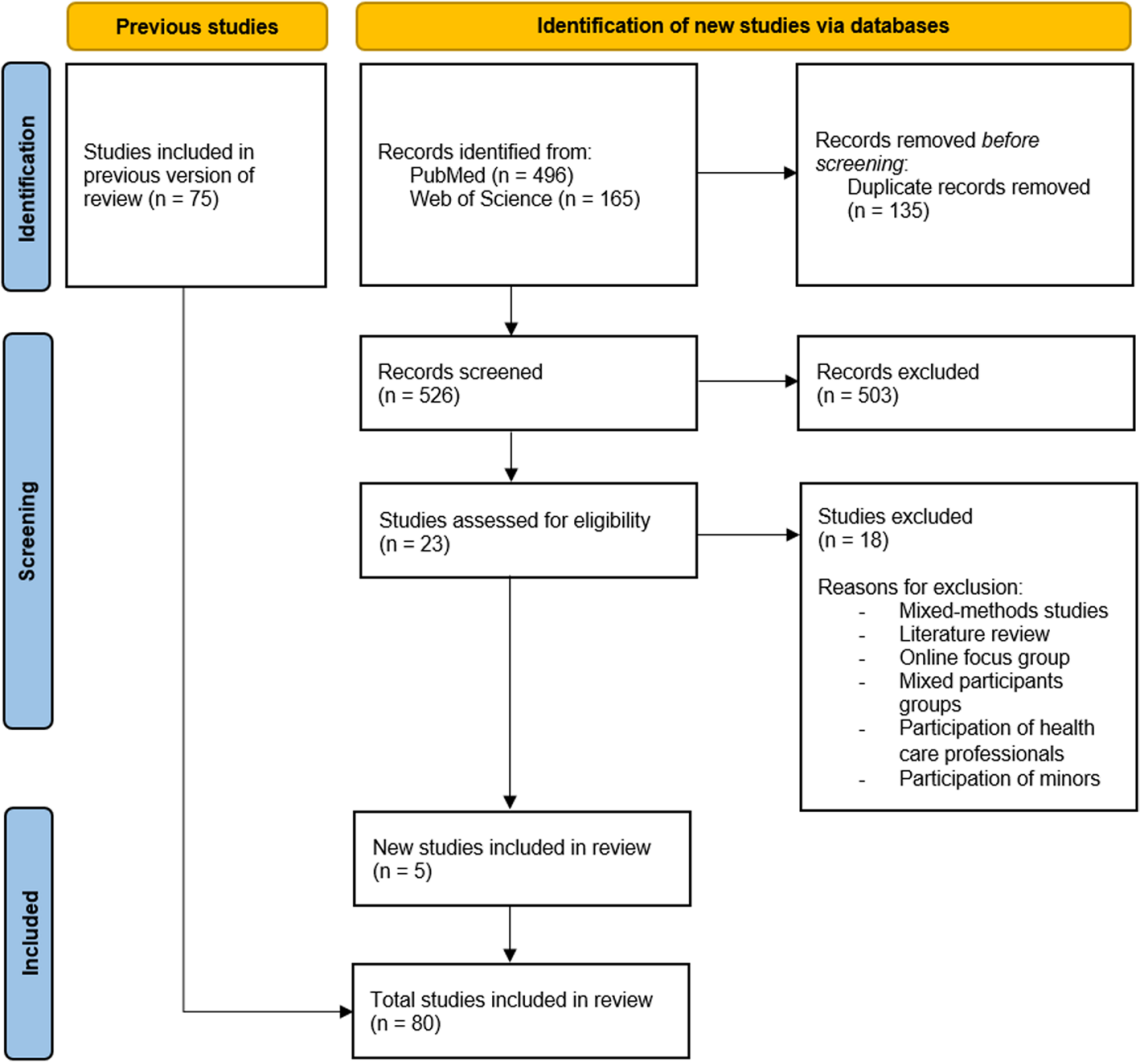
PRISMA flowchart for update search

For both blocks, relevant synonyms of the respective key terms were used as free text to enable a comprehensive search. In PubMed, we also integrated relevant medical subject headings. As no controlled vocabulary is available for Web of Science, we used a text word search there. In addition, limitations regarding the publication language and publication date were integrated directly into the respective search strategy. The complete search strategies for both databases can be found in Additional file 1; Fig. 1 summarises the search procedure and the selection process. The search was conducted on 31 January 2023 and updated on 06 November 2024 (Fig. 2), with the subsequent screening of all hits taking place over a four-week period from February to March 2023 and in November 2024. Subsequently, a duplicate clean-up was carried out with Citavi 6.

### Selection process

The title/abstract screening to identify potentially relevant studies was initially performed independently by two investigators (CJ, SW) for 10% of the total hits according to the inclusion criteria. Differing decisions were discussed by the authors. If no consensus could be reached, the publication in question was initially considered for full-text screening to make a decision based on the information provided here. Screening of the next 10% of the total hits was continued until the deviation rate between the individual assessments was no more than 5%. Screening of the remaining hits was performed by one person (CJ) and, in case of ambiguity, one of the other authors was consulted to reach consensus. If no agreement could be reached, the publication in question was first included in the full-text screening so that a decision could be made on the basis of further information.

Next, the full-text screening was carried out. For this purpose and to allow for a manageable amount of data, we took two steps: first, we assigned random numbers to all studies preliminary included from the title/abstract screening. Then, we screened the full text of each study starting with study “1” and continued this process until 75 studies were identified as eligible (initial search). Initially, 10% of this sample was screened independently by two persons using the inclusion criteria to create a common understanding of inclusion and exclusion criteria (CJ, SW). Conflicts were discussed and agreed upon. If no consensus could be reached, a third person was consulted (JL). The screening of the remaining studies in the random sample was carried out by one author (CJ). Uncertainties were discussed with an additional person and a decision was subsequently made (SW, JL). Both the title and abstract screening and the full-text screening were performed using Rayyan. For the update search, the title/abstract screening remained the same; for the full-text screening, we screened all potentially eligible studies due to the small number of identified publications.

### Data collection

Data extraction for each included study was performed using a previously developed extraction table with the following information:

- Bibliographic information (first author, title, year of publication, language of publication, country, etc.)
- Characteristics of the study participants (target group, number, age)
- Characteristics of the recruiters / research team (who, level of experience)
- Recruitment planning (use of literature/best-practice examples, sampling aim)
- Recruitment conduct: where, how, when, by whom
- Quality assurance and additional reporting details (use of incentives, patient/public involvement, limitations, conflicts of interest, use of reporting guidelines)

To extract the data, we first developed a common understanding of the extraction process: three researchers individually extracted relevant text passages for a joint sample of 10% of the included studies, excluding the update search (*n* = 5). We then compared the extracted data and discussed differences. Then, CS continued to extract the data for all of the remaining studies and agreed all ambiguities or ambiguous text passages with an additional person (SW, JL).

### Data analysis

After we had extracted any relevant information into the extraction table (Additional file 2), we assigned all text passages in all extraction categories a keyword/code to narrow down the information as far as possible. Then, the data was quantified using absolute and relative frequencies in order to identify patterns and trends between the included studies, and to help us structure the results section. For instance, we listed all methods that were used to inform about the possibility to participate in an FG study, and added the frequency for each. In addition, we collected narrative examples for each extraction category to be used to illustrate the results. This process was initially performed by three researchers for 10% of the findings (CJ, SW, JL) to create a common understanding, and then continued by CJ for the remaining data. Any case of unclear coding or summary was highlighted and discussed with another researcher. Finally, we summarized the results narratively for each of the assessment categories [32]. This included a descriptive (frequency and percentages) overview for each individual item (e.g. how many studies described a sampling aim), followed by indicating which of these aspects were well described/reported and which not, and providing qualitative details and examples to illustrate how each assessment item was described in the original publications included in this study (see also Table 2).

**Table 2:** Examples for the reporting of recruitment

Recruitment aspect	Example	In-text reference
Sampling target—quantitative	"Recruitment targeted 15 patients per group. This accounted for expected attrition rates of 20 to 50%; while 8–10 patients is the optimal number of attendees for focus group participation.”	E1
Sampling target—qualitative	"The goal of recruitment was to assemble a diverse group of participants, with regard to age and gender, from neighbourhoods that had reported poor sleep, which the GIS mapping had suggested were predominantly African American majority areas.”	E2
Use of literature/ guidelines	“Three to five FGs with five to eight participants in each setting are recommended in the literature (…).”	E3
Sample size planning	"Our sample size was based on the anticipated number of patients required to reach thematic saturation on the concept of cough symptom severity (…).	E4
Selection process	"We purposefully worked with maximum variation in sampling [according to Braun & Clarke (2013)]; that is, we sought diversity in sex, age and types of cancer as well as stages in the disease trajectory to include needs that arise in different types of cancer and at different moments in the trajectory"	E5
Experience with recruitment	"The research group consisted of four Swedish-born researchers without a migrant background, two women and two men. (…) the group included a senior professor, an associate professor, a senior researcher and a Ph.D. student, with experience of both research and practice in the fields of health promotion, migration, health literacy and rehabilitation. (…) one of the researchers had extensive experience of qualitative research. Two of the researchers have extensive knowledge and experience [regarding] health literacy."	E6
Inclusion criteria	“Patients eligible for CTS were adults aged 18 to 80 years who were admitted to the ICU for > 2 days or had a total injury burden reflected in a New Injury Severity Score (NISS) of ≥ 16 and survived to discharge."	E7
Recruitment places	„Participants were recruited from the outpatient clinics of Hadassah Medical Centre and the primary clinics of Meuhedet Health Services clinics of the Meuhedet Health Services.“	E8
Recruitment methods	“IYA members recruited the reseach participants (…) by using the word of mouth as well as networks within community structures. The research team then contacted women (…) to participate in focus group discussions.”	E9
Recruitment staff	“A Community Advisory Board (CAB) was established to launch the research process. The CAB consisted of a total of 24 community members who were recruited via direct correspondence from either MCA representatives or the research team.”	E10
Incentives	“The flyers noted that each participant would be provided with a twenty-dollar gift card to a local grocery and/or retail store.”	E11
Participatory planning of recruitment	"Participants were recruited with a range of strategies based on the principles of CBPR (…) The academic and community partners developed recruitment materials with community residents"	E12
Reaching specific groups	"Due to the community partnership, the researchers were able to hold focus groups with hard-to-reach participants; those who cannot speak English fluently or would prefer to communicate in Somali, and those who would be hesitant to participate in the research without the community partner's involvement."	E13
Recruitment limitations	“Limitations include conducting online focus groups which excludes those experiencing digital poverty. (…) More broadly, most Malawian youth live in rural areas and may not have access to social media or internet so they would not have seen the study advertisement.”	E14

## Results

### General information on the included studies

From the PubMed and Web of Science search (total *n* = 2360 hits after removal of duplicates, including the update search), we identified *n* = 158 potentially relevant FG studies in the initial search based on the abstract/title screening. Following our pre-defined screening process, *n* = 75 studies (49%) meeting the inclusion criteria were selected for analysis. In the update search, we identified *n* = 23 studies as potentially relevant, all of which were assessed in full text, resulting in *n* = 5 additional included studies. In total, n = 80 studies were included in the final analysis. The majority of these were published in 2018–2019, i.e. prior to the pandemic, which caused challenges for conducting studies with face-to-face meetings among researchers and participants. A total of 61% of the studies originate from the USA, Canada, Australia and the UK, a further 39% from countries without English as the main language, for example Netherlands, Germany and Denmark (Additional file 3). Diabetes, cancer, mental health, addiction and chronic diseases were addressed most frequently.

### Planning the recruitment

#### Defining a sampling target

To map the spectrum of opinions, perspectives, etc. for the respective study topic, 16 studies (20%) specified a concrete sampling target, either via the number of focus groups to be conducted (e.g. *n* = 8) [33], or specified the number of study participants required (e.g. Rapport et al. 2019: Table 2, E1) [34]. In very few cases, the studies did not state ‘basic’ quantitative indicators such as a specific group size as a goal, but rather focused on qualitative aspects such as the composition of the participants (e.g. Sonnega et al. 2019: Table 2, E2) [35].

#### Use of literature and guidelines

**Fig. 3:**
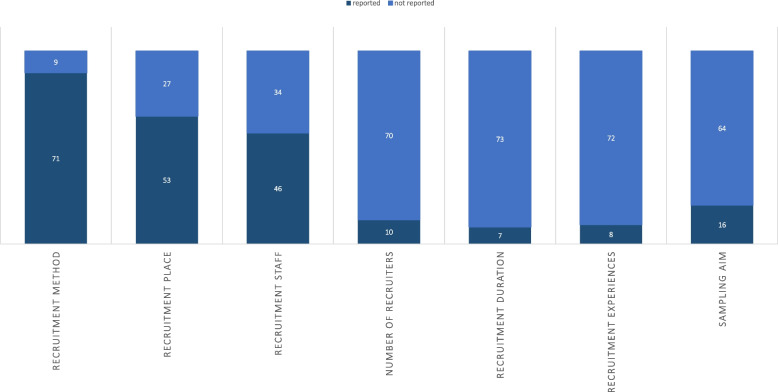
Reported aspects of recruitment, part A

For a more precise, ‘better’ planning of the required study participants or the sampling target, 5 of the included studies (6%) indicated that previously published studies served as orientation [36]. Here, they mainly referred to the planning of the group size, for example Kum et al. 2022 (Table 2, E4) [37] and, in few cases, defined the principles for the selection process more specifically (e.g. Linn et al. 2019: Table 2, E5) [38]. The remaining studies (*n* = 75) did not mention having used specific guidelines for planning and conducting qualitative research (see Fig. 3 for a numerical overview of this part).

Nevertheless, 12 of 80 studies (15%) stated they had used reporting guidelines to present the methods and findings and hence, covered the basic, fundamental aspects regarding where the recruiting was done, how and who participated in the studies eventually (COREQ, SQRQ).

#### Research team experience

In the included studies, there were isolated references to the extent to which the research team already had previous experience in planning and conducting qualitative research (n = 8; 10%), such as in Mårtensson et al. 2020 (Table 2, E6) [39].

### Implementation of the recruitment

#### Recruitment criteria

Looking at the inclusion criteria for the sample, many of the studies often stated 2 (*n* = 25), 3 (*n* = 32) or 4 (*n* = 14) criteria (range: 1–6). The most frequently applied criteria included a specific national language (23%), nationality (13%) and a specific disease (20%), with many studies specifying more than one criterion (88%) [40]. In addition, the studies varied considerably in the total number of people included (7–202) — for which the number ranged from 20–40 participants in 29% of cases and from 40–70 participants in 36%.

#### Recruitment settings

We were able to identify 27 different recruitment locations from the 80 studies. Participants were most often recruited in hospitals, doctors’ surgeries or outpatient clinics (*n* = 27; 34%) [41]. Digital recruitment channels were reported much less frequently (*n* = 11; 15%). Six studies (8%) stated that participants were recruited in mosques, churches and other everyday locations. In individual cases, the search also took place at universities (*n* = 3; 4%), nursing homes (*n* = 4; 5%) and via the researchers’ personal network (*n* = 6; 8%). However, no more detailed information was available for the latter.

#### Recruitment methods

The most frequently used methods of recruitment include approaching potential study participants directly (*n* = 38; 48%) or distributing flyers (*n* = 19; 24%). Digital approaches, such as recruitment emails and online advertising, were mentioned in 12 of 80 (15%) and 17 of 80 (21%) cases respectively — the latter includes advertising via social media such as Facebook and LinkedIn, advertising on websites, as well as newsletters and apps [42]. In 11 cases (14%), researchers approached suitable people by telephone about participating in the study or FG. In 4 cases (5%), it was reported that recruitment was based on the snowball sampling method, meaning recruited participants invited people from their circle of acquaintances to take part in the study.

#### Recruiter characteristics

With regard to the persons responsible for recruitment, in the largest proportion of the 80 studies, the recruitment was carried out by those who were directly involved in the study, i.e. research project staff (*n* = 17, 21%). In 13 cases (16%), it was reported that medical staff, such as doctors, nurses and practice staff, drew patients’ attention to the study participation as part of their treatment services [43]. Occasionally, studies also referred to collaboration with community partners (*n* = 8; 10%), contact persons for a network (*n* = 4; 5%) or patient organisations (*n* = 3; 4%); in two cases, a recruitment company was commissioned to identify potential study participants. The number of people involved in recruitment was specified in 10 studies (13%). In most cases, one to two people were involved in this task. Exceptions include Ali et al. (2022), who used a community advisory board consisting of 24 community members for recruitment [43].

#### Recruitment period

Furthermore, regarding the recruitment period, only 7 out of 80 (9%) studies specified a period that, in these cases, spanned three to six months.

### Strategies to facilitate recruitment

#### Use of incentives

**Fig. 4:**
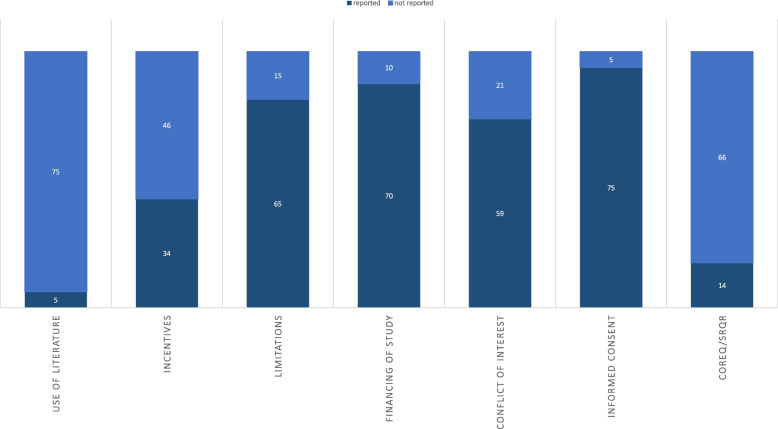
Reported aspects of recruitment, part B

In almost half of the studies (*n* = 34; 43%), the researchers used incentives to increase the attractiveness of participation (see Fig. 4 for a numerical overview of the remaining parts). The majority of these incentives were financial allowances of between 10–100 dollars (dollar was the currency in all studies that provided this information), with compensation most frequently set at 20–50 dollars (*n* = 9; 11%) [35]. In a further 14 studies (18%), the researchers awarded gift cards or vouchers also worth 20–50 dollars after the focus groups. In two studies (3%), the authors stated that they had covered the travel costs for the participants and two others reported that catering was provided for the participants.

#### Participatory approaches

Overall, 15 of the 80 included studies (19%) mentioned having incorporated principles of participatory research into their own work, for instance during the planning phase (e.g. Sonnega et al. 2019: Table 2, E12) [35]. In other cases, working with the community allowed the researchers to recruit participants for the FG who are normally difficult to reach, such as in the case of Linney et al. 2020 (Table 2, E13) [44]. In the 15 cases that reported participatory approaches, however, respective elements were not limited to the recruitment phase, but were also used, for example, for the subsequent dissemination of the results.

### Evaluation of recruitment

A total of *n* = 58 limitations relating to recruitment were mentioned in 65 of 80 studies (81%) (see Fig. 3). These included a sample size that was too small (*n* = 8; 10%), a biased selection of participants (*n* = 29; 36%), and limited results (*n* = 7; 9%). In 14 cases (18%), it was also mentioned that the sample did not allow the results to be generalized or that the samples were random (*n* = 2; 3%), although qualitative research does not typically aim for generalization.

## Discussion

The aim of our meta-research study was to investigate what information published focus group studies in health research report on the planning and conduct of recruitment of study participants, and what this information reveals about the comprehensiveness and thoroughness of the actual recruitment practices. We undertook this study because focus groups are currently a widely used research method, but to our knowledge there is little insight into their participant recruitment practices. To do this, we randomly selected 80 publications from a systematic literature search and used a descriptive scoring matrix that included key aspects of the recruitment process. Our findings can be used to improve the reporting of this part of conducting (qualitative) research, and can also help health researchers to think carefully about the different steps of recruitment, thereby improving the quality and validity of research.

### Reporting of recruiting

Overall, our findings suggest that to date there is neither structured nor consistent reporting of recruitment plans and procedures in published FG studies in health research — with considerable variation in reporting across publications. Firstly, this stands in contrast to the available information in reporting guidelines as well as recruitment literature, which detail crucial aspects that may be considered in terms of ‘best practice’ in reporting, but also actually conducting the recruitment: study participant characteristics (e.g. clearly specifying and delimiting a target group), research team characteristics (e.g. considering whether the research team has been sufficiently trained in recruitment and if not, providing insights into fundamental aspects), recruitment planning (e.g. summarizing previous recruitment strategies that proved successful and how they relate to the intended study), recruitment conduct (e.g. providing clear arguments to potential participants for how their participation will be valuable), quality assurance (e.g. involving members of the target group in the recruitment planning and conduct).

While aspects related to the characteristics of FG participants and information on who recruited them, where and how are fairly often available, a number of other aspects are regularly missing. These include details of the people involved in approaching potential study participants, the use of literature and guidelines for (better) planning, the skills and experience of the research team in recruitment, the duration of recruitment, details of sampling objectives, and the use of incentives to attract participants.

The use of guidelines for reporting qualitative research, such as COREQ and SRQR, is also rarely mentioned in the studies we analysed, despite their widespread recognition and relevance in the scientific community. Both of these well-known guidelines in this field cover questions of participant selection — which indeed should not be confused with actual guidance or instructions about which steps to take — to some extent, particularly regarding which participants were selected, how many, and with what methods. However, our results show that there are several additional elements that could be considered when planning and conducting the recruitment, such as who is responsible for each task, and how potential participants are motivated to take part in a study. As noted above, these aspects are comparatively underreported, suggesting areas where reporting practices could be improved to provide a more comprehensive view of recruitment strategies. Reporting guidelines such as COREQ and SRQR could address this by detailing such aspects more explicitly, and/or incorporating findings from studies that have summarized good practices / evidence for successful recruitment. Additionally, actors such as journal editors and peer reviewers may point authors more explicitly towards its use. For instance, journals could integrate respective information as part of the requirements for information on the research methods in ‘preparing your manuscript’ sections. However, as we found, reporting guidelines are not yet applied rigorously, so recruitment would not necessarily improve as long as this is the case. On the other hand, the various considerations and options to plan and implement recruitment comprehensively may require separate instructions. Hence, while some attempts have been made, e.g. by umbrella organizations such as Cochrane [3] or individual research groups [12], a widely known and approved/agreed recruitment guideline does not seem to exist in health research. Apart from creating such overarching documents, we suggest that key recruiting principles could be incorporated in the training of researchers at their host institutions, for instance in clinical research courses or workshops on qualitative research methods often provided by health science faculties. In addition, our paper can serve as a key resource guiding researchers on essential recruitment considerations to report in future focus group studies, thereby improving the consistency and comprehensiveness of recruitment reporting in health research.

### Methods used for recruiting

Beyond simply reporting the above information, our findings suggest that the measures taken to recruit FG participants vary considerably in terms of methodological detail and depth. For example, while there is a range of recruitment channels and methods overall, many studies tended to recruit participants in a relatively unilateral and ‘obvious’ way, e.g. through personal contact with patients during appointments at a doctor’s surgery or hospital. Furthermore, the main responsibility and workload seemed to lie with the researchers — and, where relevant, with the health care staff. As a result, there was comparatively little collaboration with other potentially helpful actors, such as local councils or social care agencies. Finally, the studies we analysed often did not consider the different ways of increasing the interest and willingness of potential participants to participate in a FG: financial remuneration is only one reason among many for people to participate in research [5, 45]. However, for example, carefully weighing up distinct recruitment channels for their effectiveness or cooperating with people and institutions who naturally have direct relationships/contact with the study’s target group may contribute to conducting research both more efficiently (in terms of the available resources) and effectively (finding the ‘right’ participants).

Previous research on participant recruitment in health research has focused on identifying common barriers and facilitators, such as the experience and skills of research teams (e.g. [46]). On the one hand, our study is likely to be consistent with key findings, such as the fact that recruitment is more successful when researchers engage more personally with potentially interested study participants [3] and when the reasons for and aims of a particular study are described comprehensibly [47]. On the other hand, while much seems to be known in theory about what can facilitate or hinder recruitment, our study suggests that — at least in relation to recruitment for FGs — this knowledge is not yet being thoroughly applied in recruitment practice. It is also be possible that researchers may not even be aware of the various conclusions and/or recommendations that have been made. If this is the case, then future research or practice initiatives should focus on efforts to communicate and translate findings on this topic to those responsible for planning and conducting recruitment. This would also include the need to generate evidence on how researchers actually approach the relevant steps. Further, it seems important to understand what kind of support they would welcome in practice, which could relate to more knowledge, guidelines on how to conduct recruitment, or better conditions to cover tasks such as approaching a wide range of diverse potential study participants.

In addition to focusing on the practices and needs of researchers, in a previous study we argued for the need to focus on the perspective of study participants, e.g. the factors that influence the decision to participate in a study or not [2]. For instance, providing a clear description in the study invitation about how one’s own participation will either be beneficial for oneself or for others has been suggested as an important aspect when approaching potential participants. Given that FGs are a specific research setting due to their ‘group’ nature, with which potential study participants may often be unfamiliar, research teams could benefit from a better understanding of how and where to approach candidates in this regard.

A particular challenge here is the aspect of engaging ‘hard-to-reach’ individuals and communities in research [47–49]. In our study, we found little or no evidence that researchers consider the need for specific strategies or additional efforts to reach such individuals, e.g. those who are typically more reluctant to consider research participation — even though this may be very beneficial to increase diversity in the perspectives of study participants, thereby uncovering important aspects necessary for providing comprehensive answers for a certain research question. This may constitute a good example of the importance of providing recruitment training for researchers, which could draw researchers’ attention to developing strategies for collaborating for instance with local community organizations during the recruiting phase. However, further research into the extent to which hard-to-reach people are considered for FG may be warranted, not least because our approach only looked at published studies.

### Participatory approaches for recruiting

Focusing on the perspective of study participants relates to a rather peripheral issue explored in this paper, namely fostering the active involvement of the target group in study. There is now a wealth of work that has not only provided arguments for how patient and public involvement (PPI) can benefit research, and which methods can be used at which stages of the process and for which purposes [50–52], but also ongoing discussion about the extent to which PPI is now a regular part of research, how to overcome the various practical challenges such as avoiding tokenistic involvement, and how to generate evidence of its precise benefits to the research process (see for example [53]). In our case, there was some evidence of PPI activities alongside the planning and running of FGs, with about 1 in 5 studies referring to this approach. However, these rare references to PPI may also confirm the difficulties and questions associated with implementing a more thorough, regular PPI practice, not least because the few reports do not help to generate more evidence about how it contributes to ‘better’ research. In fact, recruiting for FGs is a good opportunity to make research more participatory, as approaching study participants through PPI contributors can be considered a realistic, manageable task in the sense that peers often have better access to certain target groups than researchers. The benefits of participatory approaches to FG recruitment also seem likely to apply to other aspects addressed in this study, such as developing compelling arguments for why participation is worthwhile, and identifying additional recruitment channels that in turn contribute to sample diversity.

### Limitations

Although we used a detailed approach to identify and analyse published FG studies, our research has a number of limitations. Firstly, to keep the work feasible, we did not include all eligible publications identified during the search, which would have contributed to an even larger sample. This is also true for the language restriction, but differences in the methodological approaches to recruitment are unlikely to occur according to where the study has been conducted. Since we randomly selected the studies, it is unlikely that more or different studies would have led to very different findings. Further research may still be relevant to investigate recruitment for online focus groups, e.g. to understand whether the method of data collection also impacts the method of recruiting participants. Also, our results are based on the information reported in the publications, and this information may not always be complete. To be certain, it would have been necessary to interview or survey corresponding authors about their recruitment practices. Nevertheless, there are basic, often mandatory, requirements for methodological details to be provided in a publication, and researchers are generally aware of what information they should provide as part of qualitative research. Therefore, it cannot be assumed that authors ‘hid’ major parts of how they actually recruited FG participants. By assessing not only whether a particular aspect of recruitment was reported or not, but also what was mentioned, we were able to make substantive findings.

## Conclusion

Focus groups are a well-established and important part of health research, not least because this method can reveal the extent to which individuals have different (or shared) perspectives on complex issues such as ‘what works’ and ‘what doesn’t work’. However, balancing the pros and cons of issues and/or exploring the diversity of perspectives requires a careful approach to selecting participants for the necessary interactions. The often incomplete reporting of recruitment, as well as the actual approaches taken in the studies we included in our sample, suggest that this methodological aspect can often not be considered well justified and comprehensive, even though recruitment already involves a lot of effort by research teams. We therefore conclude that guidelines designed to assist researchers, such as COREQ for reporting qualitative research or published recruitment recommendations (e.g. [3, 4, 47]) for actual planning and implementation, are not yet adequately serving their purpose. In addition, researchers may benefit from more practical support, such as early training on key principles and options for effective recruitment strategies, provided by institutions in their immediate professional environment, e.g. universities, faculties or scientific associations. In order to inform such potential interventions, further research is needed to generate evidence on the preferences of those who are responsible for recruiting study participants.

## Supplementary Information

Additional file 1. Search strategy.

Additional file 2. Data extraction sheet.

Additional file 3. Overview of included studies.

## Data Availability

The data extraction sheet is provided as part of the additional files. The data extracted from the studies is available in an open access format with a CC BY 4.0 license here: https://doi.org/10.26068/mhhrpm/20241106-000.

## Abbreviations

FG: Focus groups
PPI: Patient and public involvement
COREQ: Consolidated criteria for the reporting of qualitative research
SRQR: Standards for reporting qualitative research
