# Supplementary material for: Recruiting participants for focus groups in health research: a meta-research study
Source: BMC Med Res Methodol. 2025 Jan 14;25:9. doi: 10.1186/s12874-025-02464-x (PMC11730470; doi:10.1186/s12874-025-02464-x)
Supplement: Supplementary file 1 — Additional file 1. Search strategy. [file 12874_2025_2464_MOESM1_ESM.docx]

Additional file 1 – Search strategy

PubMed

| **Search** | **Query** | **Results** |
| --- | --- | --- |
| #1 | Health Services Research[mh] OR Public Health Systems Research[mh] | 185,964 |
| #2 | “health services research”[tiab] OR “public health systems research”[tiab] OR “health research”[tiab] OR “health care research”[tiab] OR “healthcare research”[tiab] | 33,776 |
| #3 | #1 OR #2 | 214,108 |
| #4 | Focus groups[mh] | 35,186 |
| #5 | “focus group*”[tiab] OR “group discussion*”[tiab] OR “discussion group”[tiab] | 68,590 |
| #6 | #4 OR #5 | 74,123 |
| #7 | #3 AND #6 | 7,244 |
| #8 | #7 AND (“2018” [Date – Publication] : “2022”[Date – Publication]) | 1,739 |
| #9 | #8 AND ((english [Language]) OR (german [Language])) | 1,728 |

WebOfScience

| **#** | **Search Query** | **Results** |
| --- | --- | --- |
| #1 | TS= (“health services research” or “public health systems research” or "health research” or “healthcare research” or “health care research”) | 31,296 |
| #2 | TS= ("focus group$" or "group discussion$" or "discussion group$”) | 79,856 |
| #3 | #1 and #2 | 935 |
| #4 | #3 and LA= (English or German) | 927 |
| #5 | #4 and DOP= (2018-2022) | 453 |
