# Supplementary material for: Recruiting participants for focus groups in health research: a meta-research study
Source: BMC Med Res Methodol. 2025 Jan 14;25:9. doi: 10.1186/s12874-025-02464-x (PMC11730470; doi:10.1186/s12874-025-02464-x)
Supplement: Supplementary file 2 — Additional file 2. Data extraction sheet. [file 12874_2025_2464_MOESM2_ESM.docx]

Additional file 2: Data extraction sheet

Part A – Bibliographic information

| # | First author | Year | Country | Journal |
| --- | --- | --- | --- | --- |
| 1 |  |  |  |  |
| 2 |  |  |  |  |
| 3 |  |  |  |  |
| 4 |  |  |  |  |
| 5 |  |  |  |  |
| … |  |  |  |  |
| … |  |  |  |  |
| 75 |  |  |  |  |

Part B – Reporting of recruitment process

| # | Target group |  | Number of participants |  | Participant age |  | Recruitment duration |  | Recruitment method |  | Recruitment channel |  |
| --- | --- | --- | --- | --- | --- | --- | --- | --- | --- | --- | --- | --- |
|  | Rele-vant info | Summary | Relevant info | Summary | Relevant info | Summary | Relevant info | Summary | Relevant info | Summary | Relevant info | Summary |
| 1 |  |  |  |  |  |  |  |  |  |  |  |  |
| 2 |  |  |  |  |  |  |  |  |  |  |  |  |
| 3 |  |  |  |  |  |  |  |  |  |  |  |  |
| 4 |  |  |  |  |  |  |  |  |  |  |  |  |
| 5 |  |  |  |  |  |  |  |  |  |  |  |  |
| … |  |  |  |  |  |  |  |  |  |  |  |  |
| … |  |  |  |  |  |  |  |  |  |  |  |  |
| 75 |  |  |  |  |  |  |  |  |  |  |  |  |

Part B - continued

| Study title | Recruitment staff (who) |  | Recruitment staff (how many) |  | Sampling aim |  | Use of literature |  | Recruitment experience |  | Incentives |  |
| --- | --- | --- | --- | --- | --- | --- | --- | --- | --- | --- | --- | --- |
|  | Relevant info | Summary | Relevant info | Summary | Relevant info | Summary |  |  |  |  |  |  |
| 1 |  |  |  |  |  |  |  |  |  |  |  |  |
| 2 |  |  |  |  |  |  |  |  |  |  |  |  |
| 3 |  |  |  |  |  |  |  |  |  |  |  |  |
| 4 |  |  |  |  |  |  |  |  |  |  |  |  |
| 5 |  |  |  |  |  |  |  |  |  |  |  |  |
| … |  |  |  |  |  |  |  |  |  |  |  |  |
| … |  |  |  |  |  |  |  |  |  |  |  |  |

Part C – Additional information

| Study title | Patient-public involvement |  | Study limitations |  | Study financing |  | COI |  | Consent |  | Reporting guideline |  |
| --- | --- | --- | --- | --- | --- | --- | --- | --- | --- | --- | --- | --- |
|  | Relevant info | Summary | Relevant info | Summary | Yes | No | Yes | No | Yes | No | Yes | No |
| 1 |  |  |  |  |  |  |  |  |  |  |  |  |
| 2 |  |  |  |  |  |  |  |  |  |  |  |  |
| 3 |  |  |  |  |  |  |  |  |  |  |  |  |
| 4 |  |  |  |  |  |  |  |  |  |  |  |  |
| 5 |  |  |  |  |  |  |  |  |  |  |  |  |
| … |  |  |  |  |  |  |  |  |  |  |  |  |
| … |  |  |  |  |  |  |  |  |  |  |  |  |
