# Supplementary material for: Recruiting participants for focus groups in health research: a meta-research study
Source: BMC Med Res Methodol. 2025 Jan 14;25:9. doi: 10.1186/s12874-025-02464-x (PMC11730470; doi:10.1186/s12874-025-02464-x)
Supplement: Supplementary file 3 — Additional file 3. Overview of included studies. [file 12874_2025_2464_MOESM3_ESM.docx]

Additional file 3 – overview of studies

| First author, year | Objective | Target group | Topic |
| --- | --- | --- | --- |
| Mollard, 2024 | inform the overall research study  design, refine and tailor a strengths-focused intervention on overcoming Black  maternal health disparities, and build relationships with the Black community | Black or African american of any sex | Maternal health |
| Robles, 2023 | explore the determinants of healthy aging | Older black american community | Healthy aging |
| Sachdeva, 2024 | explore acceptability and perspectives of women in Dschang regarding usage of a screening tool for cervical cancer relying on artificial intelligence | Women aged 30-49 with previous participation in cancer screening programme | AI for cervical cancer screening |
| Hellingman, 2023 | explore the perspectives of patients with CRLM regarding e-consultation of transmural specialists | Patients diagnosed with colorectal cancer | Colorectal cancer |
| Wanigaratne, 2023 | describe the drivers of son preference; understand experiences of son preference among Punjabi-Canadians; identify and co-design an educational tool | Punjabi Canadians | Culturally competent health care |
| Abdel-Rahman,  2022 | understand what is important for people with type 2 diabetes, to identify relevant aspects for Patient-Reported Outcome Measures in diabetes care | people with type 2 diabetes | diabetes |
| Abuelmagda,  2019 | explore the experiences of immigrant kurdish patients in norway related to the management of type 2 diabetes | people with type 2 diabetes | diabetes |
| Adams, 2018 | assess how marginalization in the mainstream US society, as measured by acculturation and race, contributes to differences in patients’ subjective experiences and responses to prescription drugs | patients who had been recently prescribed a prescription drug | prescription drugs |
| Ali, 2022 | explore perspectives of Muslims on the role of the mosque community in supporting community members mental health needs, barriers to mental health care | muslims | mental health |
| Alyah, 2019 | explore the quality of maternal-fetal and newborn antenatal care, delivery, and postnatal care services in Jordan | pregnant and postpartum women | maternal-child care |
| Amutah-Onukagha, 2018 | gain insight from women living with HIV on how to improve Project THANKS | african american women with substance abuse disorders | HIV/AIDS |
| Asquith, 2021 | assess barriers and facilitators to participation of transgender and gender diverse patients in clinical research | transgender and gender diverse patients | clinical research |
| Ayers, 2018 | examine Marshallese mothers’ beliefs, perceptions, and experiences of prenatal care and potential barriers | marshallese mother | prenatal care |
| den Bakker, 2019 | gather participants’ experiences with full recovery in different treatment phases of multimodal treatment and to identify their needs during these phases | cancer survivors | cancer |
| Barkin, 2021 | determine the potential acceptability of home visiting services within this vulnerable population | women with high-risk pregnancies | high risk pregnancy |
| Biswas, 2018 | explore the perception of communities in Bangladesh the common practices used, and health-seeking behaviors sought after a burn injury | burn survivors and ther relatives | burn injury |
| Black, 2021 | understand patients’ cancer center experiences, explore racial differences and inform systems-level interventions | cancer patients | cancer health disparities |
| Bolliger, 2022 | follow a approach to identify work stressors as they are perceived by office worker | officeWorkers | occupational stress |
| Boroughs, 2019 | inform the development of a transdiagnostic integrated treatment platform that will focus on mental and physical health outcomes | adult MSM With a History of Being Bullied | HIV |
| Beirne, 2021 | Understand how work is experienced at the community level and to identify community interventions to establish a culture of healthy work | community residents | precarious work |
| Rawson, 2018 | explore the feasibility and willingness of citizens to engage in strategic decision making, | citizens | infection research |
| Livingston, 2020 | adress unmet need for posttrauma center care | trauma survivors | trauma |
| van der Wilk, 2018 | provide insight into the ideas of ankle-foot orthosis users on the importance of activities and suggestions for an improved ankle-foot orthosis design | ankle-foot orthosis users | ankle-foot orthosis |
| Muthulingam, 2019 | inform a tool by describing and rank-ordering patients’ considerations when deciding whether to start medication | patients Seeking Treatment for Opioid Disorder | opioid use disorder |
| Lokossou, 2021 | explore socio-cultural practices and their influence on feeding practices of mothers and their children | mothers | feeding  practices |
| Wallace, 2021 | examine the experiences of pain and discrimination and stigma across diverse marginalized communities | people living with  pain | chronic pain |
| Concannon, 2020 | explore how patients view heterogeneity in trials and whether they can participate in methodological  discussions about this concept. | patients | patient-centered research |
| Mpondoa, 2018 | investigate the what, when and how sexual health communication occurs in rural South African families | women (18-35 years old) | sexual health |
| Magez, 2018 | explore the evaluation of experiences with integrated care from the perspective of mental ill patients | mental ill people | psychiatric care |
| Sawyer, 2021 | create a community-led, theory based dissemination plan to engage local Black sexual minority men in an active dissemination process | black, cisgender  sexual minority men | dissemination |
| LeBlanc, 2022 | collect data from a sample of TGD community health center patients on health research priorities to inform future TGD-centered research | transgender and gender diverse (TGD) people | transgender |
| Mortensen, 2018 | explore the long-term health related quality of life and support needs in MBC patients of all ages in the Danish context | metastatic breast cancer patients (MBC) | breast cancer |
| Kulkarni, 2019 | explore what clinical and social outcomes of glaucoma surgery are important to patients. | glaucoma patients | glaucoma |
| Valandra, 2019 | explore domestic violence perceptions of African Americans with heterogeneous backgrounds and experiences of violence | african americans | domestic violence |
| Daffin, 2021 | identify themes of risk and resilience for long-term outcomes among young adults diagnosed with JFM in childhood | young adults who are diagnosed with JFM | juvenile-onset Fibromyalgia |
| Rezaie, 2020 | determine the unique post-discharge needs of Iranian women diagnosed with severe mental illness | women with severe mental ilness | mental ilness |
| Spook, 2019 | identify worker needs and preferences regarding the use of sensor technology applications in the workplace | workers with  physically demanding work | physically demanding work |
| Villarreal‐Garza, 2019 | describe clinical and information needs, identify unmet support services and guide interventions | young breast cancer patients in Mexico | breast caner |
| Kum, 2022 | develop a new patient-reported outcome measure addressing cough severity. | patients with refractory/ unexplained chronic cough | chronic cough |
| Kim, 2018 | explore single mothers’ experiences with social services policies for their independent living | single mothers | childcare |
| Sonnega, 2019 | understand more about the sleep experiences of residents of an urban community reporting sleep insufficiency | participants over 18 years | sleep deficits |
| Garcia, 2022 | describe recommendations for correcting persistent areas of mismatch in the adapted Conexiones program | latina mothers | cancer |
| Tan, 2018 | explore the experiences of older Singaporeans with Type 2 diabetes in diabetes self-care management | older adults with type 2 diabetes | diabetes |
| Edmondson, 2022 | use a participatory research approach to capture what healthy lifestyle means to people who are diagnosed with SMI | people diagnosed with SMI (serious mental illness) | care planning |
| Skea, 2022 | understand public attitudes and social value in regard to the baby box scheme | parents | baby boxes |
| Molina, 2018 | address gaps in the literature via analysis of qualitative needs assessment in counties with relatively high rates of cancer incidence, morbidity, and mortality | rural adult female residents | cancer |
| Mårtensson, 2020 | explore experiences and needs concerning health related information for newly arrived refugees in Sweden | arabic and somali speaking refugees | health information |
| Navarro-Millán, 2019 | understand perspectives patients on electronic communication and patient reported outcome data collection | patients with rheumatoid arthritis (RA) | rheumatoid arthritis |
| Rajah, 2021 | explore the sources of emotional distress, the related support and the unmet needs of cancer survivors | cancer patients from diverse backgrounds | cancer |
| Jumbe, 2022 | share our experience of engaging youth in Malawi through advocacy organisations to inform cultural adaptation of a mental health literacy intervention | young people | mental  health literacy |
| Gonzales, 2018 | explore perspectives on biological monitoring in  environmental health research | members of Zuni Pueblo | biological monitoring |
| Herrewegh, 2018 | assess the suitability and comprehensibility of a newly developed urological patient-reported outcome measurement “Uromate.” | patients with a overactive bladder syndrome | urology |
| Kwan, 2018 | identify quality of life domains and subdomains relevant to patients with different spondyloarthritis subtypes | patients with SpA | psoriatic arthritis |
| Rebitschek, 2019 | explore information needs and attitudes in regard to epigenetic risk assessment of female cancers | women | cancer |
| Ibsen, 2019 | explore perspectives of patients with low back pain on how to qualify a patient centred consultation | patients with low back pain | back pain |
| Linney, 2020 | explore community beliefs and views about the causes of mental illness, treatment, and access to medical services | somali participants | mental illness |
| López-Entrambasaguas, 2020 | explore the perceptions of chronic patients from the day-care center toward subjective health needs that are not being met by the socio-health system. | people with chronic diseases | chronic diseases |
| Elm, 2019 | understand sources of stress among midwest american indian adults | american indian adults with type II diabetes | diabetes |
| Estrada, 2018 | gain the views regarding what they believe would support them and their peers in visiting the dentist regularly | racial/ethnic minority older adults | oral health |
| Larson, 2021 | explore perceived risk, and safety issues of household insecticides: | residents of the Alto Selva Alegre | insecticides |
| Bosma, 2020 | explore the experiences of workers with a chronic condition & identify existing barriers,possible support needs for staying at work | workers with one or more chronic conditions | chronic condition |
| Brubacher, 2021 | sewing as strategy for arts-based inquiry in research, situated within that highlighted Nunavut Inuit women’s childbirth experiences | pregnant women | arts-based research |
| Sayer, 2019 | perspectives on diet and physical activity with serious mental illness | urban african americans | mental illness |
| Moukam, 2021 | understand the barriers affecting women’s decision-making process regarding participation in a cervical cancer screening program | female patients and their partners | cancer |
| Zøylner, 2019 | explore patients’ and relatives’ experiences with the surgical breast CPP and to identify any unmet needs. | patients who had surgery for breast cancer, and relatives | cancer |
| Tint, 2018 | explore the service experiences of women with autism spectrum disorder | women with autism spectrum disorder | autism |
| Lunney, 2018 | obtain patient-identified factors related to satisfaction with epilepsy surgery | epilepsy surgery patients | epilepsy |
| Grieb, 2021 | identify community-developed strategies that may enhance community engagement in research with BSMM. | black sexual minority men (BSMM) | HIV/STI |
| Hawarden, 2020 | identify important areas for future osteoporosis research | experience of osteoporosis | osteoporosis |
| Kearns, 2021 | reflect on the recruitment strategy and outcomes, and suggest direction for future study and implementation | Māori | asthma |
| Muzyamba, 2018 | exploring the relevance of community mobilization in the promotion of maternal health care in resource-poor settings | women living with HIV | HIV |
| Tiilikainen, 2019 | examines older people’s perceptions of quality of life from the perspective of access and use of health and social care services | older people living alone. | social care |
| Nakajima, 2022 | gain understanding about attitudes toward mental health and addition, and perspectives about health research | somali immigrants | mental health |
| Nunn, 2018 | assess knowledge nakai about HIV and the HIV care continuum | african american pastors and ministers | HIV |
| Lal, 2019 | gain an in-depth understanding on the subject of relapse of young people receiving services for a first-episode psychosis | family members | mental health |
| Linn, 2019 | provide an overview of intervention guidelines on how to address patients' practical needs for support in expressing instrumental concerns and emotions | cancer patients and survivors | cancer |
| Rapport, 2019 | examine perceptions of hospital care | inpatients at SVHA | patient experience |
| Mosor, 2021 | explore whether PROMs commonly used in inflammatory arthritis adequately cover the perspective of young people from different European countries | young people with  different arthritis forms, | arthritis |
| Hughes, 2020 | inform the development of a novel programme for mental health by first eliciting their positive and negative prior experiences with mental health provider | young adults with depression | mental health |
| Smith, 2019 | examines cancer survivors’ perspectives about sharing PGHD with central cancer registries | metastatic breast cancer survivors | cancer |
